# Views of Ethical Best Practices in Sharing Individual-Level Data From Medical and Public Health Research: A Systematic Scoping Review

**DOI:** 10.1177/1556264615594767

**Published:** 2015-07

**Authors:** Susan Bull, Nia Roberts, Michael Parker

**Affiliations:** 1 University of Oxford, UK

**Keywords:** biomedical research ethics, data sharing, data release, data access, research data, research governance, low-income countries, middle-income countries, clinical research, health policy, privacy, systematic review

## Abstract

There is increasing support for sharing individual-level data generated by medical and public health research. This scoping review of empirical research and conceptual literature examined stakeholders’ perspectives of ethical best practices in data sharing, particularly in low- and middle-income settings. Sixty-nine empirical and conceptual articles were reviewed, of which, only five were empirical studies and eight were conceptual articles focusing on low- and middle-income settings. We conclude that support for sharing individual-level data is contingent on the development and implementation of international and local policies and processes to support ethical best practices. Further conceptual and empirical research is needed to ensure data sharing policies and processes in low- and middle-income settings are appropriately informed by stakeholders’ perspectives.

Policies mandating the sharing of individual-level data from biomedical and public health research are becoming widespread and commanding increasing support from large funding bodies, regulatory agencies, and the pharmaceutical industry (Medical Research Council, 2011; National Institutes of Health, 2003; Nisen & Rockhold, 2013; Research Information Network, 2008; Toronto International Data Release Workshop Authors 2009; UK Data Archive, 2011; Walport & Brest, 2011; Wellcome Trust, 2009). Discussions of data release in the literature highlight the importance of taking seriously both ethical arguments for sharing individual-level data from health research and the need to develop appropriate governance and protections (Antman, 2014; Eichler, Petavy, Pignatti, & Rasi., 2013; Greenhalgh, 2009; White, 2013; Zarin, 2013).

The increasing amount of clinical and public health research being conducted in low- and middle-income settings has the potential to generate datasets of significant value to researchers seeking to address disease burdens in such settings (Manju & Buckley, 2012). Consequently, there is a pressing need to determine how best to develop effective, ethical, and sustainable approaches to data sharing in such contexts. Experiences of data release for genomic research suggest that challenges raised by individual-level data sharing in low- and middle-income settings will be different in important and morally significant ways from those arising in high-income settings (Parker et al., 2009). In particular, although timely data sharing may be particularly important in low- and middle-income settings to inform effective and urgently needed public health interventions, it is important that data sharing is conducted in a way that does not disadvantage or harm researchers, research institutions, communities, and participants in such settings. Potential benefits and harms of data sharing are discussed in more detail below and summarized in Table 1.

**Table 1.** Summary of Potential Benefits of and Concerns About Data Sharing.

Reasons to share individual-level data	Concerns about sharing individual-level data
To improve science Enable verification, replication, and expansion of research results Address biases, deficiencies, and dishonesty in research Enable novel analyses and increase study power Improve meta-analyses Maximize data use, particularly for datasets that cannot be replicated Inform research design and research funding Improve teaching resources Increase primary data producers’ academic profiles and collaboration opportunities	May hamper science Reputational harms of critical secondary analyses Consequences of flawed/poor quality secondary analyses Reduction of incentives for primary research Increased incentives to conduct short-term research rather than long-term research Opportunity costs of curating and sharing data
To improve health Inform health care planning and allocation Inform regulatory review Improve evidence base for clinical decision making Improve use of health care resources Improve patient care	May hamper health Effects of flawed secondary analyses on scientific evidence base Burden of evaluating validity of secondary analyses Effects of second-guessing regulatory procedures, policies, and processes
Explicit moral claims Importance of maximizing the value and utility of data Promotion of scientific values Promotion of best practices in research conduct, analysis, and reporting Demonstration of respect for research participants Promotion of the public good	Explicit ethical issues Protection of participants’ privacy and confidentiality Validity of consent, including broad consent Potential harms of secondary research for research participants including discrimination and stigma Researchers’ ability to fulfill commitments made to research participants during data collection Effects of moral distance and limited awareness of the context in which data were collected Potential impacts on public trust and confidence of conflicting analyses Balancing the interests of differing stakeholders in data sharing Making best use of limited research resources
	Barriers to sharing Costs of developing and maintaining appropriate expertise and infrastructure Curation costs Ownership, intellectual property rights, and commercial confidentiality Lack of policies and processes

## Potential Advantages of Data Sharing

Sharing individual-level data from clinical and public health research can be valuable in multiple ways. Sharing data allows for independent scrutiny of research results to ensure they are reliable and reproducible, and increases the accountability of researchers (Estabrooks & Romyn, 1995; Godlee & Groves, 2012; Kuntz, 2013; Manju & Buckley, 2012; Mello et al., 2013; Sieber, 2006). This may be particularly important where there are differing approaches to analyses (Smith, 1994) or where there are concerns that reports of research have been selective, biased, or dishonest (Doshi, Goodman, & Ioannidis, 2013; Gotzsche, 2011b; Rathi et al., 2012; Rodwin & Abramson, 2012; Ross, Gross, & Krumholz, 2012). Sharing data also enables identification of gaps in research and can inform both future research priorities and research design (Eichler et al., 2013; Gotzsche, 2011a; Sandercock, Niewada, Czlonkowska, & International Stroke Trial Collaborative Group, 2011; Strech & Littmann, 2012). Some datasets of particular value may not be able to be re-collected due to changes in available treatment and disease incidence, and may be useful as reference datasets, particularly in different contexts, such as low- and middle-income settings (Eichler et al., 2013; Sandercock et al., 2011).

Many commentators have discussed the value of conducting novel analyses with shared datasets, including testing innovative statistical methods and alternative analytical approaches (Coady & Wagner, 2013; de Wolf, Sieber, Steel, & Zarate, 2005; Hrynaszkiewicz & Altman, 2009; Pisani & AbouZahr, 2010; Toronto International Data Release Workshop Authors, 2009; Vickers, 2006; Whitworth, 2010). Meta-analyses combining individual-level datasets may provide more reliable results than those based on summary data (Chan et al., 2014; Pisani & AbouZahr, 2010). Meta-analyses may also provide different results from the primary studies and permit examination of topics such as the heterogeneity of treatment effects, subgroup effects, temporal and geographical effects, and identification of rare safety events (Anderson & Merry, 2009; Chan et al., 2014; Dawson & Verweij, 2011; Manju & Buckley, 2012; Mello et al., 2013).

Additional arguments in favor of sharing data are that it can be an efficient and cost-effective means of maximizing the utility of a dataset for research purposes and for teaching and methodology development (Gotzsche, 2011b; Manju & Buckley, 2012; Smith et al., 2014; Walport & Brest, 2011). Increasing use of collected data can also reduce unnecessary duplication of research, which in turn limits potential harms to and burdens on research participants (Eichler et al., 2013; Rani, Bekedam, & Buckley, 2011; Strech & Littmann, 2012).

These claims suggest that data sharing can make a very important contribution to public health, by improving the evidence base used to make regulatory, funding, and clinical decisions, and to make the best use of available resources (Hrynaszkiewicz & Altman, 2009; Hughes, Wells, McSorley, & Freeman, 2014; Rathi et al., 2012; Rodwin & Abramson, 2012; Ross, Lehman, & Gross, 2012). As a routine best practice in research, it may contribute to improving public faith in research and drug regulation, particularly by promoting accountability and transparency in processes where there are potential conflicts of interest (Haines & Gabor Miklos, 2011; Hampton, 2011; Rani et al., 2011; Zarin, 2013).

In addition to the potential of advancing scientific development and health, commentators have discussed ethical imperatives for promoting data sharing. Principles of fairness and reciprocity require data be shared to benefit communities that fund research indirectly and that provide the data on which research relies (Langat et al., 2011; Pisani & AbouZahr, 2010; Strech & Littmann, 2012; Tangcharoensathien, Boonperm, & Jongudomsuk, 2010; Walport & Brest, 2011). In addition, respect for research participants requires that their contributions to research be maximized by making the best use of their data. In particular, expectations that the results of research will be disseminated to advance science must be honored (Gotzsche, 2011b; Mello et al., 2013; Pisani & AbouZahr, 2010; Walport & Brest, 2011).

## Potential Disadvantages of Data Sharing

Numerous concerns and issues about sharing individual-level health research data have been discussed in the literature in addition to potential benefits. A core concern is to ensure that the privacy of participants is protected during secondary uses of data (Castellani, 2013; de Wolf, Sieber, Steel, & Zarate, 2006a; Eichler et al., 2013; Nisen & Rockhold, 2013; Savage & Vickers, 2009; Walport & Brest, 2011; Zarin, 2013). Processes for de-identifying data must be not only robust but also proportionate if the utility of the data is to be preserved (Antman, 2014; de Wolf, Sieber, Steel, & Zarate, 2006b; Eichler et al., 2013). Concerns have been raised about the ability of primary researchers to guarantee that re-identification will not take place (Mello et al., 2013), particularly when reverse engineering and/or the combination of datasets may increase chances of identifying specific participants (Estabrooks & Romyn, 1995; Geller, Sorlie, Coady, Fleg, & Friedman, 2004; Nisen & Rockhold, 2013; Rabesandratana, 2013; Wieseler, McGauran, Kerekes, & Kaiser, 2012).

Although curating and sharing data may make the most efficient and effective use of datasets, preparing data for research and implementing appropriate policies and processes require significant effort, expertise, and resources (Anderson & Merry, 2009; Mello et al., 2013; Rathi et al., 2012; C. T. Smith et al., 2014; Walport & Brest, 2011). Lack of resources needed to share data has been identified as an impediment to data release in empirical research in higher income settings (Mello et al., 2013; Rathi et al., 2012; Reidpath & Allotey, 2001; Savage & Vickers, 2009; C. T. Smith et al., 2014) and as a serious obstacle in low- and middle-income settings (Manju & Buckley, 2012; Pisani & AbouZahr, 2010; Rani et al., 2011; Whitworth, 2010).

Concerns have been raised that if sufficient safeguards are not in place, inappropriately prepared or shared data may hamper, rather than promote, public health (Nisen & Rockhold, 2013; Pearce & Smith, 2011; Piwowar, Becich, Bilofsky, Crowley, & on behalf of the caBIG Data Sharing and Intellectual Capital Workspace, 2008; Spertus, 2012). Data may be misinterpreted, or the subject of biased, inappropriate, or poorly designed studies (Greenhalgh, 2009; Kirwan, 1997; Pisani, Whitworth, Zaba, & Abou-Zahr, 2010a; Rathi et al., 2012; Spertus, 2012; Wieseler et al., 2012). The results of such studies may mislead health care providers and regulators, lead to false hopes or unfounded concerns about treatments, reduce public confidence in research, and result in litigation (Anderson & Merry, 2009; Castellani, 2013; Kuntz, 2013; Mello et al., 2013; Nisen & Rockhold, 2013; Ross & Krumholz, 2013). In addition, incentives for novel biomedical research may be reduced, if secondary data users can “free-ride” on the efforts of those collecting the data (Castellani, 2013; Langat et al., 2011; Rabesandratana, 2013; Rathi et al., 2012; Ross & Krumholz, 2013; Zarin, 2013).

## Stakeholders’ Interests in Data Sharing

Sharing individual-level research data will affect the interests of stakeholders in different ways. Primary researchers have interests in conducting initial analyses of data they have collected, and in receiving appropriate acknowledgment for dataset production (Castellani, 2013; Lopez, 2010; Pisani & AbouZahr, 2010; Tangcharoensathien et al., 2010; Whitworth, 2010). Research participants and the communities from which they are drawn have interests in understanding that data may be shared, the consequences of sharing, and ways in which potential harms of sharing can be minimized (Mello et al., 2013; Pearce & Smith, 2011; Piwowar et al., 2008). Research funders have interests in promoting the utility of datasets and may also have interests in commercial exploitation of research results (Anderson & Merry, 2009; Castellani, 2013; Eichler et al., 2013; Kmietowicz, 2013; Mello et al., 2013).

Data sharing policies and process must recognize and respond to the differing interests of stakeholders appropriately if they are to effectively promote the benefits of data sharing and minimize potential harms. Calls have been made for policies and processes for data sharing to be informed by, and developed in consultation with, relevant stakeholders (Manju & Buckley, 2012; Vallance & Chalmers, 2013; Whitworth, 2010). This scoping review sought to map evidence about stakeholders’ experiences of data sharing and their perspectives of best practices, particularly in low- and middle-income settings, with the aim of informing future policy development and research agendas (Parker & Bull, 2015).

## Method

Scoping reviews seek to identify literature relevant to the research objective and may include a variety of research formats and conceptual literature (Arksey & O’Malley, 2005; Armstrong, Hall, Doyle, & Waters, 2011). This study sought to review published literature on stakeholders’ experiences of sharing individual-level data from medical and public health research and views of ethical best practices reported in peer-reviewed journals. Inclusion criteria for the study encompassed a broad range of article types, including empirical studies, news articles, opinion pieces, features, editorials, reports of practice, and theoretical articles. The initial search strategies for capturing views in this range of article formats were developed through an iterative process and used a combination of text words and subject headings (see Online Supplementary Materials 1 at http://jre.sagepub.com/supplemental).

The following databases were searched for relevant studies: Embase (OvidSP)[1974-present], Global Health (OvidSP)[1973-present], Global Health Library–Regional Databases (Virtual Health Library) [http://www.globalhealthlibrary.net], MEDLINE(R) In-Process & Other Non-Indexed Citations and MEDLINE(R) (OvidSP) [1946-present], ABI Inform (Proquest) [1971-current], PAIS International (Proquest)[1977-current], Science Citation Index (Web of Science Core Collections, Thomson Reuters) [1945-present] and WHOLIS (Virtual Health Library) [http://www.globalhealthlibrary.net]. The original search was conducted on June 24, 2013, and searches were repeated on December 9, 2013, and June 27, 2014, to update findings. No language or publication date limits were applied. Research relevant to low- and middle-income countries was isolated and grouped using a geographic search filter; however, all references were screened. (The full search strategy for Medline is available in Online Supplementary Materials 2).

**Figure 1.**
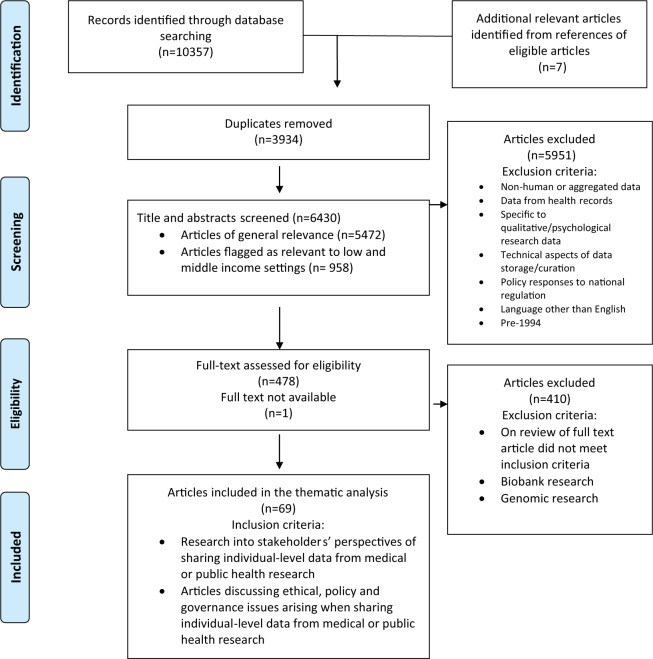
PRISMA 2009 flow diagram of the scoping review. *Source.* Moher, Liberati, Tetzlaff, Altman, and the PRISMA Group (2009).

The total of 6,430 abstracts identified by the strategy were screened, 958 of which were flagged as being particularly relevant in low- and middle-income settings. A matrix of inclusion and exclusion criteria was developed to inform screening (see Figure 1).

All abstracts were reviewed by a single researcher, with sample of 20% of abstracts being co-reviewed by additional researchers using a trial outline of inclusion and exclusion criteria. After co-reviewing 20% of abstracts, the value of a dual review was assessed. Given the complexity of consistently determining from abstracts which articles contained discussions of relevant ethical, policy, and governance issues, and the large number of abstracts to be screened, multiple review of all the abstracts was considered inefficient. Instead, a single reviewer applied revised inclusion and exclusion criteria consistently, marking articles that were potentially relevant (228), and additional articles that required full text review to determine relevance (246). References from these two categories were imported into bibliographic software (Endnote X6), which was then used to track decisions during a detailed review (King, Hooper, & Wood, 2011).

In scoping reviews, to ensure appropriate identification of the literature, it may be important to adopt an iterative approach to study selection (Arksey & O’Malley, 2005; Armstrong et al., 2011). Following screening of full text articles, five empirical studies of stakeholders’ perspectives of sharing individual-level data from clinical and public health research were identified, all of which reported views and practices of researchers and research institutions from high-income settings. During full-text screening, articles focusing on samples and individual-level data from biobanks and genomic research were not routinely excluded, particularly when they reported on perspectives from data subjects or from stakeholders in low- and middle-income settings. A subsequent review of ethical, policy, and governance issues raised in such papers demonstrated some important differences with issues raised by sharing data from clinical and public health research, and they were subsequently excluded from the review.

The full text of the final 69 shortlisted papers was imported into qualitative data analysis software (NVIVO 10; see Table 2). Descriptive codes were developed to chart perceived advantages of data sharing, barriers and concerns about data sharing, and recommendations for best practices in governing data sharing.

**Table 2.** Articles Included in the Scoping Review.

Type of Article	Articles of general relevance	Articles of particular relevance in low-and middle-income settings
Empirical research articles	Kirwan, 1997; Rathi et al., 2012; Reidpath & Allotey, 2001; Savage & Vickers, 2009; C. T. Smith et al., 2014.	
Articles focusing on ethics, policy, and governance issues	Anderson & Merry, 2009; Antman, 2014; Brewer, Potterat, & Muth, 2010; Castellani, 2013; Chan et al., 2014; Coady & Wagner, 2013; Dawson & Verweij, 2011; de Wolf, Sieber, Steel, & Zarate, 2005, 2006a, 2006b; Doshi, 2013; Doshi, Goodman, & Ioannidis, 2013; Editorial, 2014; Eichler, Petavy, Pignatti, & Rasi, 2013; Estabrooks & Romyn, 1995; Geller, Sorlie, Coady, Fleg, & Friedman, 2004; Godlee & Groves, 2012; Goldacre, 2013; Gotzsche, 2011a, 2011b, 2012; Greenhalgh, 2009; Haines & Gabor Miklos, 2011; Hampton, 2011; Harris, 2011; Hawkes, 2012, 2013; Hede, 2013; Hrynaszkiewicz & Altman, 2009; Hughes, Wells, McSorley, & Freeman, 2014; Kmietowicz, 2013; Kuntz, 2013; Langat et al., 2011; Lopez, 2010; Mello et al., 2013; Nisen & Rockhold, 2013; Pearce & Smith, 2011; Piwowar, Becich, Bilofsky, Crowley, & on behalf of the caBIG Data Sharing and Intellectual Capital Workspace, 2008; Rabesandratana, 2013; Rodwin & Abramson, 2012; Ross, Gross, & Krumholz, 2012; Ross, Lehman, & Gross, 2012; Ross & Krumholz, 2013; Sandercock, Niewada, Czlonkowska, & International Stroke Trial Collaborative Group, 2011; Sieber, 2006; G. D. Smith, 1994; Sommer, 2010; Spertus, 2012; Strech & Littmann, 2012; Toronto International Data Release Workshop Authors, 2009; Vallance & Chalmers, 2013; Vickers, 2006; Walport & Brest, 2011; White, 2013; Wieseler, McGauran, Kerekes, & Kaiser, 2012; Zarin, 2013	Manju & Buckley, 2012; Pisani & AbouZahr, 2010; Pisani, Whitworth, Zaba, & Abou-Zahr, 2010a; Pisani, Whitworth, Zaba, & AbouZahr, 2010b; Rani, Bekedam, & Buckley, 2011; Sankoh & Ijsselmuiden, 2011; Tangcharoensathien, Boonperm, & Jongudomsuk, 2010; Whitworth, 2010

## Results

This section begins by reviewing empirical research into stakeholders’ experiences of, and views about, best practices in sharing individual-level data from medical or public health research. It then outlines the views expressed in articles focusing on ethical, policy, and governance issues arising when sharing such data. It concludes by focusing on issues identified as particularly relevant to best practices when sharing data from low- and middle-income settings.

### Empirical Research

There is very limited empirical research into stakeholders’ experiences of sharing individual-level data from clinical or public health research, and their views about best practices when doing so. This review identified five empirical studies, all of which sampled researchers and reviewers from high-income settings. Details of the studies, including the primary findings reported in the original articles, are set out in Table 3.

**Table 3.** Empirical Research Into Stakeholders’ Experiences and Perspectives.

Author, publication year	Study aim	Sample	Methods	Key findings
Kirwan, 1997	Determine opinions of making original data available for alternative analysis.	21 (84%) of 25 invitees from pharmaceutical companies with an interest in rheumatology. Locations of respondents not discussed.	Letter with brief rationale for sharing data and asking whether it would be useful for trial data related to a publication to be shared via a databank and if so, what issues might arise.	5 (24%) of respondents were in favor of sharing data via a databank and 16 (76%) were not. The four most common reasons for not sharing data were: it is inappropriate to conduct analyses not defined in the protocol, authors may be willing to share data for a specific purpose on request instead, other researchers may conduct inappropriate analyses due to a lack of awareness of aspects of the original study, and further analysis is *post hoc* and data dredging. 12 (57%) participants thought difficulties might arise with sharing trial data due to commercial sensitivity extending beyond the point of publication.
Reidpath and Allotey, 2001	Investigate the preparedness of researchers to share their data.	21 (72%) of 29 inquiries sent to corresponding authors of research articles in the *British Medical Journal* Locations of respondents not discussed.	Emailed specific request to reanalyze the data used in a published study (15) or a general inquiry about willingness to share data from a published study(14).	9 (60%) of authors receiving specific requests and 12 (86%) of authors receiving general requests responded. Of the 21 responding authors, one shared the dataset and one was prepared to share the dataset without further conditions, 10 were prepared to release the data in principle subject to further discussions/conditions, three would not release data (suggesting they conduct new analyses themselves or that sufficient data were available in the articles), and six were ultimately non-committal. Authors wanted to know more about why the data were being requested and the proposed analyses. Some authors wanted more time to conduct their own analyses before sharing data, some required conditions to be met (such as payment or contracts to be completed) and others needed to consult with co-investigators prior to release.
Savage and Vickers, 2009	To test effects of journal policies requiring data to be shared.	10 requests for datasets from corresponding authors of research articles in PLoS Medicine or PLoS Clinical Trials Locations of respondents not discussed.	An emailed request for a dataset to test a pre-specified hypothesis about prediction modeling.	Two authors had changed institution and could not be contacted. One author asked for further details and then shared the dataset. Four authors declined to share the data. When reminded of the journal policy, one said that a formal request to the research group was required, two said it was too much work, the fourth said that he was not permitted to share the data and wouldn’t have published in the journal if he’d known it was a requirement. Three authors didn’t respond to the first email, two of whom didn’t respond to a second email and the third declined to share data because more analyses were proposed.
Rathi et al., 2012	To investigate clinical trialists’ opinions and experiences of sharing data with non-collaborating investigators.	317 (46%) of 683 corresponding authors of clinical trials published in 2010 or 2011 in one of the six highest impact general medical journals. Respondents were from the United States or Canada—167 (53%) Western Europe—113 (36%) Other—37 (12%).	38 item adaptive-response online survey.	236 (74%) of respondents supported sharing de-identified data via repositories, and 229 (72%) thought investigators should be required to share data on request. 56 (18%) were required to deposit trial data in a repository by funders and 149 (47%) had received an individual request to share data. Concerns about sharing data through repositories included: potentially misleading secondary analyses, ensuring appropriate data use, ensuring clarity of data elements, indirect costs associated with sharing, colleagues’ abilities to publish original research, ability to publish own research, scientific or academic recognition, direct costs associated with sharing, protecting commercially sensitive information, obtaining consent, and maintaining confidentiality. Reasons for denying individual requests for data related to: potentially misleading analyses, potential for misinterpretation of data, potential mistrust of requestor’s intent, ability to publish own research, colleagues’ ability to publish original research, indirect costs associated with sharing, potential prohibition by formal agreement, potential lack of recognition, protecting commercially sensitive information, direct costs associated with sharing, being unsure of employer or funder policy, protection of patient confidentiality, and potential lack of consent. Reasons for sharing data in response to individual requests included: promoting new research and open science, enhancing robustness of previous research, facilitating student or fellow opportunities, avoiding redundant data collection, increasing impact of own research, professional or personal relationship with the requestor, additional academic recognition, compliance with employer or funder policy, compliance with journal policy.
C. T. Smith et al., 2014	Evaluate support and identify major issues for establishing a central repository of individual participant data.	30 (42%) of 71 reviewers affiliated with the Cochrane Collaboration’s individual participant data meta-analysis method group. Respondents were from the United Kingdom—22 (73%) Other European Countries—6 (20%) Australia—1 (3%) Canada—1 (3%).	Synopsis and link to 16 question online survey.	25 (83%) of respondents thought a central repository would be valuable, 25 (83%) would be willing to deposit data in such a repository provided conditions met. The five most commonly suggested conditions that needed to be met for deposition were: approval from primary data source, appropriate acknowledgment of primary data source, involvement of original investigators in the process, reassurance about who would access the data, and the presence of a scientific committee to review data access requests. The five most commonly expected governance arrangements were: restricted access requiring approval, data security, an oversight committee, appropriate recognition for data owners, and anonymised data.

### Best Practices in Data Sharing

In the introduction to this article, stakeholders’ views about potential benefits and harms of sharing individual-level data were outlined. When considering the implications of such potential benefits and harms for best practices in data sharing, the fundamental importance of protecting the privacy of research participants was universally acknowledged in the reviewed literature. Some authors went further and set out additional specific principles and considerations for best practices in ethical data sharing (see Table 4).

**Table 4.** Principles and Considerations to Inform Best Practices in Ethical Data Sharing.

Principles and considerations	Reference
Ensure sufficiently broad access to realize the benefits to scientific innovation and public health, which are the main justification for sharing. Ensure data are used responsibly so that poor quality analyses do not harm public health. Treatment of researchers qualified to access data must be evenhanded.	Mello et al., 2013
Data sharing processes must be accountable and transparent.	Mello et al., 2013; Rabesandratana, 2013
Equitable: The needs of researchers, secondary users, communities, and funders should be recognized and balanced. Ethical: The privacy of individuals and dignity of communities should be protected and public health promoted by productive data use. Efficient: Proportionate approaches should build on existing practice to improve the quality and value of research.	Walport & Brest, 2011
Ensure fair trade and not free trade in data.	Pisani, Whitworth, Zaba, & Abou-Zahr, 2010a; Walport and Brest, 2011
Ensure the rights and responsibilities of researchers generating data and data accessors are balanced.	Sankoh and Ijsselmuiden, 2011
Ensure the benefits of data sharing outweigh the harms, and consider whether restricting the flow of information to avoid rare adverse events is appropriate.	Vickers, 2006
Clearly specify public interests in data sharing and clearly specify any legitimate reasons to restrict access to research data (following market approval of an intervention).	Strech and Littmann, 2012
Ensure that the analytic value of the data is preserved during the protection of privacy and confidentiality.	Sieber, 2006; Vallance and Chalmers, 2013
Ensure data sharing processes are responsive to the context within which datasets were collected.	Pearce and Smith, 2011
Honor the altruism of research participants.	Zarin, 2013

### Governed Data Sharing

To maximize the potential benefits of sharing de-identified data, some stakeholders recommended that de-identified datasets should typically be made available publicly, with minimal restrictions (Doshi et al., 2013; Eichler et al., 2013; Gotzsche, 2011a, 2011b; Haines & Gabor Miklos, 2011; Harris, 2011; Ross, Gross, & Krumholz, 2012; Strech & Littmann, 2012; Vallance & Chalmers, 2013). In contrast, in the majority of reviewed papers, a governed approach to data release was considered valuable to minimize potential harms and maximize potential benefits. Some authors discussed specific advantages of adopting a governed approach to data sharing, as outlined in Table 5.

**Table 5.** Potential Benefits of a Governed Approach to Data Sharing.

Potential benefits of curation	Reference
Adequate safeguards can be established, bona fide access restrictions can be put in place.	Pisani, Whitworth, Zaba, and Abou-Zahr, 2010a; Walport and Brest, 2011
Patient privacy is increased.	Doshi, Goodman, and Ioannidis, 2013; Hawkes, 2012; Hughes, Wells, McSorley, and Freeman, 2014; Nisen and Rockhold, 2013
Poor quality research, which may lead to erroneous conclusions, can be prevented following review and requirements to adhere to a rigorous analytical plan.	Doshi et al., 2013; Eichler, Petavy, Pignatti, and Rasi, 2013; Hughes et al., 2014; Manju and Buckley, 2012; Mello et al., 2013; Nisen and Rockhold, 2013; Rathi et al., 2012
Permits compliance with legislation and or regulation.	Nisen and Rockhold, 2013
Promotes adherence to commitments made during the consent process.	Hughes et al., 2014
Enables researchers to fulfill responsibilities to ensure data are used ethically.	Pearce and Smith, 2011
Curation can be responsive to the types of data being shared. Differing approaches can be taken to aggregate and individual-level data, particularly valuable or sensitive datasets, and analyses that require detailed data that could potentially identify participants.	Geller, Sorlie, Coady, Fleg, and Friedman, 2004; Hrynaszkiewicz and Altman, 2009; Rabesandratana, 2013; Toronto International Data Release Workshop Authors, 2009; Vallance and Chalmers, 2013

To guide governed data sharing, stakeholders made a number of recommendations about appropriate policy development. The current lack of policies or inconsistent policies in some settings was considered both frustrating and inefficient, as well as providing loopholes for researchers who did not want to share data (Manju & Buckley, 2012). A number of papers recommended that harmonized policies with broad applicability be developed, following consultation with a broad range of stakeholders, including policy makers, researchers, patients, patient advocates, privacy experts, funders, research institutions, journal editors, ethicists, NGOs, and governments (Estabrooks & Romyn, 1995; Hrynaszkiewicz & Altman, 2009; Manju & Buckley, 2012; Mello et al., 2013; Vallance & Chalmers, 2013; Whitworth, 2010). These could be complemented by institutional policies where appropriate (Manju & Buckley, 2012; Piwowar et al., 2008). Areas to be addressed in the policies are outlined in Table 6. Some commentators questioned the effectiveness of guidelines and policies encouraging data sharing to date (Gotzsche, 2012; Mello et al., 2013; Savage & Vickers, 2009; Vickers, 2006) and suggested legal requirements for data sharing be implemented (Gotzsche, 2012; Vickers, 2006).

**Table 6.** Priority Areas for Policy Development.

Areas for policy development	References
Appropriate analytic methods, data and meta-data standards, including means of preserving privacy	Kuntz, 2013; Mello et al., 2013; Pisani & AbouZahr, 2010; Rani, Bekedam, and Buckley, 2011; Vickers, 2006
Determining where, how, when, and which data are archived and made available	Manju and Buckley, 2012
Determining for which trials data will be shared, which data and supporting documents will be available, the process for data sharing, how transparent the process will be, who will get access, what types of analyses are permitted, who will decide, what criteria will be used, and what ongoing role the trial sponsor might have.	Zarin, 2013
Methods to permit evaluation of individual applications, including to ensure that the use does not harm participants and is in conformity with ethical approvals	Eichler, Petavy, Pignatti, and Rasi, 2013; Toronto International Data Release Workshop Authors, 2009
Transparent, explicit, and reasonable criteria for case by case decision making	Mello et al., 2013
Requirements and rewards for the collection and curation of datasets for sharing	Pisani, Whitworth, Zaba, and Abou-Zahr, 2010a; Walport and Brest, 2011

### Best Practices in Sharing Data From Low- and Middle-Income Settings

In both the discussion of potential advantages and disadvantages of data sharing in the introductory section of this article, and the discussion of perspectives about best practices above, the views of authors discussing data sharing in low- and middle-income settings were similar to those expressed in the more substantial body of literature from higher income settings. In contrast to lower and middle-income settings, articles from higher income settings had more discussion about ways in which these issues had been addressed and data shared to date. When discussing how to provide resources for best practices in data sharing, and how to balance the interests of stakeholders in the data sharing process (particularly those generating datasets), views remained similar, but some different emphases also emerged.

The importance of balancing the interests of primary researchers and secondary data users has received considerable attention in the reviewed literature. Stakeholders from higher and lower income settings commented on the importance of ensuing that researchers received appropriate recognition for producing datasets in the subsequent publications by secondary analysts, in professional assessments, and in funding applications (Kuntz, 2013; Manju & Buckley, 2012; Pisani & AbouZahr, 2010; Pisani et al., 2010a; Piwowar et al., 2008; Rani et al., 2011; Rathi et al., 2012; Ross & Krumholz, 2013; G. D. Smith, 1994; C. T. Smith et al., 2014; Walport & Brest, 2011; Whitworth, 2010). Perspectives on authorship differed. Some commentators suggested that co-authorship or at least the chance to publish an associated response or commentary should be offered to the researchers who produced the dataset (Pearce & Smith, 2011; Savage & Vickers, 2009; Vickers, 2006). Others noted that the contribution of data creators may not be sufficient to warrant co-authorship of the secondary analysis (Anderson & Merry, 2009; Gotzsche, 2011b).

Although some commentators considered the value of releasing data prior to publication (Toronto International Data Release Workshop Authors, 2009), others noted the value of exclusive fair use periods for researchers in higher and lower income settings (Geller et al., 2004; Gotzsche, 2011b; Manju & Buckley, 2012; Pearce & Smith, 2011; Pisani & AbouZahr, 2010; Pisani et al., 2010a; Rathi et al., 2012; Ross, Lehman, & Gross, 2012; Savage & Vickers, 2009; Tangcharoensathien et al., 2010; Vickers, 2006). Such periods ranged from 12 months from the end of data collection to unspecified lengths of time, which were, in some cases, linked to the publication of an article with primary findings.

Although limited resources may be a hindrance to data sharing in higher income settings, they were identified as a very significant barrier in lower income settings (Manju & Buckley, 2012; Pisani & AbouZahr, 2010; Pisani et al., 2010a; Pisani, Whitworth, Zaba, & AbouZahr, 2010b; Rani et al., 2011; Sankoh & Ijsselmuiden, 2011; Tangcharoensathien et al., 2010; Walport & Brest, 2011; Whitworth, 2010). For high-quality individual-level data to be shared in databases with long-term sustainability, significant investment in human resources, technology, and infrastructure will be required. Training, mentoring, and career pathways need to be provided for a range of specialist support staff who will document and curate datasets and manage data release processes. Where data archives are hosted within low- and middle-income settings, expertise in managing biomedical information will be required in addition to the development of storage infrastructure.

Commentators have noted that it would be unfair to develop capacity to share data in low- and middle-income settings without also developing the capacity for data generators and secondary users from such settings to analyze that data (Pisani et al., 2010a; Sankoh & Ijsselmuiden, 2011; Walport & Brest, 2011; Whitworth, 2010). Collaboration between primary and secondary data users was discussed as a potential means of improving the quality of analyses in both higher and lower income settings (Geller et al., 2004; Kuntz, 2013; Pearce & Smith, 2011; Spertus, 2012). Stakeholders from lower income settings also focused on the value of such collaborations to build capacity among researchers generating datasets (Manju & Buckley, 2012; Pisani et al., 2010a; Tangcharoensathien et al., 2010; Whitworth, 2010).

## Discussion

The reviewed literature demonstrated considerable support for sharing individual-level data from clinical and public health research. As discussed above, numerous recommendations have been made for best practices in governing such sharing, to ensure that potential benefits are promoted and potential harms are managed appropriately. Although significant consensus about some aspects of best practice is evident, such as the need to protect the privacy of research participants, there are differences of opinion about practical achievement of these, such as the measures needed to protect privacy and the extent to which privacy can be assured (Gotzsche, 2011b; Mello et al., 2013; Nisen & Rockhold, 2013). In other areas, there is less consensus about best practices. Opinions differ, for example, about the need for and length of protected time primary researchers should have with data before they are shared (Geller et al., 2004; Toronto International Data Release Workshop Authors., 2009), and the nature of consent required, if any, for sharing de-identified data (de Wolf et al., 2005; Pearce & Smith, 2011).

Commentators have suggested that gaps and inconsistencies in policies and practices for data sharing are frustrating and inefficient, and have recommended that consensus be sought on developing harmonized policies and processes for sharing individual-level data which are informed by stakeholders’ views (Manju & Buckley, 2012; Whitworth, 2010).

This review identified just five examples of empirical literature into stakeholders’ experiences of and views about sharing individual-level data, all of which focused on the views of data producers and reviewers, primarily from higher income settings (Kirwan, 1997; Rathi et al., 2012; Reidpath & Allotey, 2001; Savage & Vickers, 2009; C. T. Smith et al., 2014). Four of the five studies have sample sizes of 30 or less, and three are five or more years old. Although the findings from these articles provide interesting insights into researchers’ opinions and practices of sharing data, some of the perspectives are dated, and differences in the research questions and approaches mean that views of best practices have not been systematically elicited.

This review was unable to identify any empirical research into research participants’ perspectives about sharing individual-level data from clinical and public health research that does not involve genetic, genomic, or biobank research. In addition, no research into stakeholders’ experiences and perspectives of best practices in sharing clinical data in low- and middle-income settings was found. To develop best practices in data sharing that are appropriate in low- and middle-income settings, empirical research into the perspectives of stakeholders from such settings is needed. We suggest that research into the perspectives of research participants, community representatives, researchers, research ethics committees, and data managers be made a priority to inform current policy development initiatives. The following five articles in this special issue begin to address this gap in the literature and report on the results of empirical studies of stakeholders’ perspectives in India, Kenya, Thailand, South Africa, and Vietnam (Cheah et al., 2015; Denny, Silaigwana, Wassenaar, Bull, & Parker, 2015; Hate et al., 2015; Jao et al., 2015; Merson et al., 2015).

Eight of the conceptual articles in this scoping review focused on the perspectives of stakeholders from low- and middle-income settings (Manju & Buckley, 2012; Pisani & AbouZahr, 2010; Pisani et al., 2010a, 2010b; Rani et al., 2011; Sankoh & Ijsselmuiden, 2011; Tangcharoensathien et al., 2010; Whitworth, 2010). These articles suggest that challenges raised by sharing individual-level data from low- and middle-income settings can differ in important and morally significant ways from those arising in high-income settings. An example is the critical importance of building capacity to generate, curate, share, and analyze high-quality datasets if data are to be shared effectively and fairly. Further theoretical analysis will be valuable to evaluate additional issues arising when sharing individual-level data in low- and middle-income settings, and to inform how best to address them (Bull, Cheah et al., 2015).

### Limitations of the Review

Although double screening of all materials is desirable in systematic reviews, it was not possible in this case due to the volume of potential references identified and the complexity of determining the relevance of papers from the supplied abstracts. To minimize error and bias, 20% of abstracts were co-reviewed, and the strategy for a structured approach to analysis was discussed by the co-authors with the collaborating partners in this study. A second limitation of this review is that it was confined to literature in peer-reviewed publications. A valuable addition to the findings of this review would be a review of policies and processes currently in place for curating and sharing individual-level data from clinical and public health research.

## Supplementary Material

Supplementary material

## Supplementary Material

Supplementary material
