# Supplementary material for: Views of Ethical Best Practices in Sharing Individual-Level Data From Medical and Public Health Research: A Systematic Scoping Review
Source: J Empir Res Hum Res Ethics. 2015 Jul;10(3):225–38. doi: 10.1177/1556264615594767 (PMC4548478; doi:10.1177/1556264615594767)
Supplement: Supplementary material [file Literature_review_JERHRE_Supplementary_materials_2_594767.pdf]

Supplementary materials 2: Medline search strategy

1 ((data or database?) and (share* or sharing or release* or releasing or disseminat* or distribut* or export* or recycl* or access*)).ti.

2 (information and (share* or sharing or release* or releasing or disseminat* or distribut* or export* or recycl* or access*)).ti.

3 (finding? and (share* or sharing or release* or releasing or disseminat* or distribut* or export* or recycl* or access*)).ti.

4 (result? and (share* or sharing or release* or releasing or disseminat* or distribut* or export* or recycl* or access*)).ti.

5 (data and research).ti.

6 Information Dissemination/

7 Access to Information/

8 ((data or database?) adj3 (share* or sharing or release* or releasing or disseminat* or distribut* or export* or recycl* or access*)).ti,ab.

9 ((data or database?) adj5 open access).ti,ab.

10 (information adj3 (share* or sharing or release* or releasing or disseminat* or distribut* or export* or recycl* or access*)).ti,ab.

11 (finding? adj3 (share* or sharing or release* or releasing or disseminat* or distribut* or export* or recycl* or access*)).ti,ab.

12 (result? adj3 (share* or sharing or release* or releasing or disseminat* or distribut* or export* or recycl* or access*)).ti,ab.

13 (data adj3 (store? or storing or storage?)).ti,ab.

14 ((data adj2 (bank* or warehous*)) or biobank* or bio-bank* or health information exchange?).ti,ab.

15 1 or 2 or 3 or 4 or 5 or 6 or 7 or 8 or 9 or 10 or 11 or 12 or 13 or 14

16 research.ti.

17 exp Biomedical Research/es, lj, mt, st [Ethics, Legislation & Jurisprudence, Methods, Standards]

18 Research/es, lj, mt, st [Ethics, Legislation & Jurisprudence, Methods, Standards]

19 *research/ or exp *biomedical research/ or *research design/

20 exp Clinical Trials as Topic/es, lj, mt, st [Ethics, Legislation & Jurisprudence, Methods, Standards]

21 exp *Clinical Trials as Topic/

22 Data Collection/es, lj, mt, st [Ethics, Legislation & Jurisprudence, Methods, Standards]

23 *Data Collection/

24 ((health or biomedical or medical or clinical or genetic* or genom* or collaborative) adj2 research).ti,ab.

25 16 or 17 or 18 or 19 or 20 or 21 or 22 or 23 or 24

26 Privacy/

27 Confidentiality/

28 Trust/

29 consensus/

30 International Cooperation/

31 Cooperative Behavior/

32 Informed Consent/

33 Consent Forms/

34 human rights/ or civil rights/ or patient rights/

35 Personal Autonomy/

36 exp intellectual property/

37 Disclosure/

38 Commodification/

39 Commerce/

40 Computer Security/

41 (privacy or confidential* or trust* or concern? or permission? or autonomy or anonym* or consent or right? or ownership or acceptab* or sovereign* or protect* or transpar* or custodian* or guardian*).ti,ab.

42 (consensus or partners* or cooperat* or co-operat* or commerc* or commodif*).ti,ab.

43 ((data or computer) adj3 (secur* or safe*)).ti,ab.

44 26 or 27 or 28 or 29 or 30 or 31 or 32 or 33 or 34 or 35 or 36 or 37 or 38 or 39 or 40 or 41 or 42 or 43

45 15 and 25 and 44

46 Ethics, Research/ or Ethics Committees, Research/

47 (ethic* or bioethic*).ti,ab.

48 ethics.fs.

49 46 or 47 or 48

50 15 and 25 and 49

51 Policy Making/ or Public Policy/ or Health Policy/ or Policy/

52 Government Agencies/ or Government/ or Government Regulation/

53 Social Control, Formal/

54 (policy or policies or regulation or regulated or mandat* or compulsory or governance or legal* or law* or "code of conduct").ti.

55 ((research or government or regulat*) adj5 (policy or policies)).ti,ab.

56 (governance or "code of conduct").ti,ab.

57 ((research or trial* or mandat* or compulsor*) adj5 (regulat* or rule* or law?)).ti,ab.

58 51 or 52 or 53 or 54 or 55 or 56 or 57

59 15 and 25 and 58

60 Information Dissemination/es, lj [Ethics, Legislation & Jurisprudence]

61 Access to Information/es, lj [Ethics, Legislation & Jurisprudence]

62 60 or 61

63 25 and 62

64 45 or 50 or 59 or 63

65 Developing Countries/

66 (Africa or Caribbean or West Indies or South America or Latin America or Central America).hw,kf,ti,ab,cp.

67 (Afghanistan or Albania or Algeria or Angola or American Samoa or Argentina or Armenia or Armenian or Azerbaijan or Bangladesh or Benin or Byelarus or Byelorussian or Belarus or Belorussian or Belorussia or Belize or Bhutan or Bolivia or Bosnia or Herzegovina or Hercegovina or Botswana or Brazil or Brasil or Bulgaria or Burkina Faso or Burkina Fasso or Upper Volta or Burundi or Urundi or Cambodia or Khmer Republic or Kampuchea or Cameroon or Cameroons or Cameron or Camerons or Cape Verde or Central African Republic or Chad or China or Colombia or Comoros or Comoro Islands or Comores or Mayotte or Congo or Zaire or Costa Rica or Cote d'Ivoire or Ivory Coast or Cuba or Djibouti or French Somaliland or Dominica or Dominican Republic or East Timor or East Timur or Timor Leste or Ecuador or Egypt or United Arab Republic or El Salvador or Eritrea or Ethiopia or Fiji or Gabon or Gabonese Republic or Gambia or Gaza or Georgia Republic or Georgian Republic or Ghana or Gold Coast or Grenada or Guatemala or Guinea or Guinea-Bisau or Guam or Guiana or Guyana or Haiti or Honduras or Hungary or India or Maldives or Indonesia or Iran or Iraq or Jamaica or Jordan or Kazakhstan or Kazakh or Kenya or Kiribati or Korea or Kosovo or Kyrgyzstan or Kirghizia or Kyrgyz Republic or Kirghiz or Kirgizstan or Lao PDR or Laos or Lebanon or Lesotho or Basutoland or Liberia or Libya or Macedonia or Madagascar or Malagasy Republic or Malaysia or Malaya or Malay or Sabah or Sarawak or Malawi or Nyasaland or Mali or Marshall Islands or Mauritania or Mauritius or Agalega Islands or Mexico or Micronesia or Middle East or

Moldova or Moldovia or Moldovian or Mongolia or Montenegro or Morocco or Ifni or Mozambique or Myanmar or Myanma or Burma or Namibia or Nepal or Netherlands Antilles or Nicaragua or Niger or Nigeria or Pakistan or Palau or Palestine or Panama or Papua New Guinea or Paraguay or Peru or Philippines or Philipines or Phillipines or Phillippines or Romania or Rumania or Roumania or Rwanda or Ruanda or Saint Lucia or St Lucia or Saint Vincent or St Vincent or Grenadines or Samoa or Samoan Islands or Navigator Island or Navigator Islands or Sao Tome or Senegal or Serbia or Seychelles or Sierra Leone or Sri Lanka or Ceylon or Solomon Islands or Somalia or Sudan or Suriname or Surinam or Swaziland or Syria or Principe or South Sudan or Tajikistan or Tadzhikistan or Tadjikistan or Tadzhik or Tanzania or Thailand or Timor-Leste or Togo or Togolese Republic or Tonga or Tunisia or Turkey or Turkmenistan or Turkmen or Tuvalu or Uganda or Ukraine or Uzbekistan or Uzbek or Vanuatu or New Hebrides or Venezuela or Vietnam or Viet Nam or West Bank or Yemen or Zambia or Zimbabwe or Rhodesia).hw,kf,ti,ab,cp.

68 ((developing or less* developed or under developed or underdeveloped or middle income or low* income or underserved or under served or deprived or poor*) adj (countr* or nation? or population? or world)).ti,ab.

69 ((developing or less* developed or under developed or underdeveloped or middle income or low* income) adj (economy or economies)).ti,ab.

70 (low* adj (gdp or gnp or gross domestic or gross national)).ti,ab.

71 (low adj3 middle adj3 countr*).ti,ab.

72 (lmic or lmics or third world or lami countr*).ti,ab.

73 transitional countr*.ti,ab.

74 65 or 66 or 67 or 68 or 69 or 70 or 71 or 72 or 73

75 64 and 74

76 (2013* or 2014*).dp,ed,yr.

77 75 and 76
